# Nutritional supplementation in children with severe pneumonia in Uganda and Kenya (COAST-Nutrition): a phase 2 randomised controlled trial

**DOI:** 10.1016/j.eclinm.2024.102640

**Published:** 2024-05-14

**Authors:** Sarah Kiguli, Peter Olupot-Olupot, Mainga Hamaluba, Elisa Giallongo, Karen Thomas, Florence Alaroker, Robert O. Opoka, Abner Tagoola, Shela Oyella, Damalie Nalwanga, Eva Nabawanuka, William Okiror, Margaret Nakuya, Denis Amorut, Rita Muhindo, Ayub Mpoya, Hellen Mnjalla, Emmanuel Oguda, Thomas N. Williams, David A. Harrison, Kathy Rowan, Andre Briend, Kathryn Maitland

**Affiliations:** a Department of Paediatrics, School of Medicine, Makerere University and Mulago Hospital Kampala, Uganda; b Busitema University Faculty of Health Sciences, Mbale Campus, Uganda; c Mbale Regional Referral Hospital Mbale, Uganda; d Kilifi County Hospital and Kenya Medical Research Institute (KEMRI) Wellcome Trust Research Programme, Kilifi, Kenya; e Clinical Trials Unit, Intensive Care National Audit & Research Centre (ICNARC), London, UK; f Soroti Regional Referral Hospital, Soroti, Uganda; g Jinja Regional Referral Hospital Jinja, Uganda; h Masaka Regional Referral Hospital Masaka, Uganda; i Department of Radiology, School of Medicine, Makerere University, P.O Box 7072, Kampala, Uganda; j Department of Infectious Disease and Institute of Global Health and Innovation, Imperial College London, London, UK; k Tampere Center for Child, Adolescent and Maternal Health Research, Faculty of Medicine and Health Technology, Tampere University and Tampere University Hospital, Tampere, Finland; l Department of Nutrition, Exercise and Sports, Faculty of Science, University of Copenhagen, Frederiksberg, Denmark

**Keywords:** Children, Africa, Pneumonia randomised controlled trial, Nutritional support, Ready to use therapeutic feeds, Anthropometry

## Abstract

**Background:**

Severe pneumonia in African children results in poor long-term outcomes (deaths/readmissions) with undernutrition as a key risk factor. We hypothesised additional energy/protein-rich Ready-to-Use Therapeutic Foods (RUTF) would meet additional nutritional requirements and improve outcomes.

**Methods:**

COAST-Nutrition was an open-label Phase 2 randomised controlled trial in children (aged 6 months-12 years) hospitalised with severe pneumonia (and hypoxaemia, SpO_2_ <92%) in Mbale, Soroti, Jinja, Masaka Regional Referral Hospitals, Uganda and Kilifi County Hospital, Kenya (ISRCTN10829073 (registered 6th June 2018) PACTR202106635355751 (registered 2nd June 2021)). Children were randomised (ratio 1:1) to enhanced nutritional supplementation with RUTF (plus usual diet) for 56 days vs usual diet (control). The primary outcome was change in mid-upper arm circumference (MUAC) at 90 days as a composite with mortality. Secondary outcomes include anthropometric status, mortality, and readmissions at Days 28, 90 and 180.

**Findings:**

Between 12 August 2018 and 22 April 2022, 846 eligible children were randomised, 424 to RUTF and 422 to usual diet, and followed for 180-days [12 (1%) lost-to-follow-up]. RUTF supplement was initiated in 417/419 (>99%). By Day 90, there was no significant difference in the composite endpoint (probabilistic index 0.49, 95% CI 0.45–0.53, p = 0.74). Respective 90-day mortality (13/420 3.1% vs 14/421 3.3%) and MUAC increment (0.54 (SD 0.85) vs 0.55 (SD 0.81)) were similar between arms. There was no difference in any anthropometric secondary endpoints to Day 28, 90 or 180 except skinfold thickness at Day 28 and Day 90 was greater in the RUTF arm. Serious adverse events were higher in the RUTF arm (n = 164 vs 108), mainly due to hospital readmission for acute illness (54/387 (14%) vs 37/375 (10%).

**Interpretation:**

Our study suggested that nutritional supplementation with RUTF did not improve outcomes to 180 days in children with severe pneumonia.

**Funding:**

This trial is part of the EDCTP2 programme (grant number RIA-2016S-1636-COAST-Nutrition) supported by the 10.13039/501100000780European Union, and UK Joint Global Health Trials scheme: 10.13039/501100000265Medical Research Council, 10.13039/501100002992Department for International Development, 10.13039/100010269Wellcome Trust (grant number MR/L004364/1, UK).

**Research in context**

**Evidence before this study**

We searched PubMed from database inception to August 1st, 2023, using the search terms “child”, “nutrition support”, “intervention”, AND “severe pneumonia”, AND “hospital” for intervention studies in children with severe pneumonia on clinical and nutrition outcomes. We had filters for English language and human studies. We found no randomised controlled trials and controlled before-and-after studies of nutrition interventions conducted in low-income and middle-income countries (LMICs). The existing evidence has largely been limited to micronutrient supplementation (zinc or Vitamin D) with no overall benefit.

**Added value of this study**

We hypothesised that children hospitalised with severe pneumonia, incorporating children across the anthropometric spectrum, supplementary feeds with RUTF, a peanut-based paste, to provide additional energy-rich, protein, fat and micronutrients to help meet the additional nutritional requirements and improve global outcomes. Our rationale considered that RUTF is widely used and if shown to benefit children following admission with pneumonia, could be rapidly deployed for nutritional support following pneumonia. We found no benefit of addition of diet-supplemented with RUTF nutritional paste in children aged 6 months to 12 years on the composite endpoint of increased mid-upper arm circumference or death at Day 90 support. Other than a marginal increase in skin fold thickness at Day 28 in the interventional arm we found no benefits on any anthropometric outcome measure. We found increase in adverse events to Day 180 (death and readmission) in the intervention arm but this may have been have been a chance finding.

**Implications of all the available evidence**

Future trials of nutritional support following pneumonia should aim to focus on the high risk undernourished group and using a feed more designed to target the metabolic needs of children with severe infection to optimise outcomes.

## Introduction

Pneumonia remains the leading cause of childhood illness and hospital admission in sub-Saharan Africa.^1^ However, outcomes remain poor including death and hospital readmission in the six months following hospital discharge.^2^^,^^3^ A prospective study, examining post-discharge mortality in Kenyan children under 5 years, found that those with anthropometric evidence of undernutrition were at greatest risk.^4^ To address the poor long-term outcomes, the integrated Global Action Plan for Pneumonia and Diarrhoea developed by the World Health Organization (WHO) and United Nations Children's Fund (UNICEF) recommends ‘continued’ feeding under the ‘Treat’ element of the Protect Prevent and Treat framework for pneumonia and diarrhoea interventions, but does not give any specific recommendations for nutritional support.^5^

Early nutritional support is commonly practiced in a wide variety of critically ill patients, including those with sepsis. Supportive nutrition is aimed at supplying vital cell substrates, antioxidants, vitamins and minerals, essential for normal cell function and to decrease hypermetabolism.^6^^,^^7^ The usual stress response to severe infection, including the increased release of catabolic hormones (cortisol and catecholamines), leads to a catabolic ‘hypermetabolic’ response in order to rapidly mobilise energy. The source is mainly from protein breakdown, leading to the conversion of amino acids (mainly alanine and glutamine) to glucose through liver gluconeogenesis. Much of the mobilised protein and amino acids are derived from skeletal muscle given its dominating abundance on whole-body protein.^8^ Consequently, there is rapid skeletal muscle breakdown leading to muscle cachexia or ‘wasting’.^6^ This has been shown to have adverse consequences in intensive care studies, even in previous healthy adults, such as delayed weaning from mechanical ventilation.^7^^,^^9^ In children surviving pneumonia with normal nutritional status or for moderate acute malnutrition (MAM), there is no current recommendation for nutritional support. We considered the potential options for nutritional supplementation including those which are given in the rehabilitation of children in high income settings, the development of a novel feed or application of an existing supplemental feed widely available in the nutrition programmes in Africa. The third option was favoured as the most likely to be generalizable were it shown to be beneficial in a clinical trial.

Current nutritional food options available in Africa in supplementary feeding using Ready-to-Use Supplementary Foods (RUSF) which in recommended for moderately undernourished children (MAM). The nutrient intakes for optimal recovery of children under 5 years with MAM were reviewed in October 2008 at a joint consultation of the WHO, UNICEF, World Food Programme and the United Nations High Commissioner for Refugees.^10^ The consensus view was that the desirable nutrient intakes in relation to energy were probably in the range between the recommended nutrient intakes for well-nourished children and the intakes recommended in the recovery phase for those with severe malnutrition.^11^ These supplementary feeds are generally only available for community nutritional programmes. The second and preferable option was Ready-to-Use Therapeutic Food (RUTF), which is a high-energy fortified food used for the follow-on nutritional rehabilitation of children hospitalised with severe acute malnutrition (SAM).^12^ We considered whether this could be extended to children at high risk of undernutrition/malnutrition. The ComPas study suggested that provision of one sachet per day (500 kcal) for children under 5 years with moderate malnutrition (defined as a mid-upper arm circumference (MUAC) 11.5 cm−< 12.5 cm) would provide 49% or more of their daily energy requirements.^13^ A study by Maust el al suggested this leads to superior outcomes.^14^

We propose that the excess post-discharge mortality associated with pneumonia may relate to the catabolic response and muscle wasting induced by severe infection and inadequacy of the diet to aid recovery.^15^ We therefore hypothesised that supplementing the usual diet of all children hospitalised with severe pneumonia, with RUTF, (a peanut-based, energy-, protein-, and fat-rich paste with added micronutrients) would help meet additional nutritional requirements and lead to decreased risk of catabolism, and improved outcomes. Our rationale considered that RUTF is widely used and, if shown to benefit children following admission with pneumonia, could be rapidly and widely deployed for nutritional support following pneumonia.

## Methods

### Study design

A Phase 2, multicentre, open-label, randomised controlled trial (COAST-Nutrition) aimed at generating preliminary data on anthropometric evidence of growth and survival to Day 90 in children receiving supplementary nutritional support in addition to usual diet (experimental) compared to usual diet alone (control). The trial was registered at ISRCTN10829073 (6th June 2018) on PACTR202106635355751 (2nd June 2021), Protocol Version 4.0 (17th February 2020). The trial protocol has been published.^16^

The trial was conducted in five sites in two countries in Africa. In Uganda, the trial enrolled children in Mbale, Soroti, Jinja and Masaka Regional Referral Hospitals. In Kenya, the trial was conducted at the Kilifi County Hospital. The trial was initially embedded, as a second randomisation at 48 h, within a clinical trial of oxygen and respiratory support, the Childrens’ Oxygen Administration Strategies Trial COAST (ISRCTN15622505) which co-enrolled up to February 2020 when the COAST trial has halted for feasibility. The results of the COAST trial were published in 2021.^17^ Following a protocol amendment (Protocol Version 4.0, 17th February 2020) the nutritional trial continued until the sample size was attained.

### Study population

Children aged between 6 months and 12 years admitted to hospital with severe pneumonia (history of respiratory illness and signs of severe pneumonia)^18^ and hypoxaemia (pulse oximetry reading of SpO_2_ <92%) who survived for 48 h were considered eligible for enrolment. Children with severe malnutrition (MUAC <11.5 cm), known chronic lung disease (not including asthma) or cyanotic congenital cardiac disease were excluded, as were children whose parent declined consent or if they had been previous enrolled in this trial. Where prior written consent from parents/legal guardians could not be obtained, ethics committees approved parental verbal assent and deferred written informed consent as soon as practicable.^19^ Otherwise, informed written consent was obtained from parents or guardians before randomisation.

### Outcome measures

The primary outcome was change in MUAC (measured by MUAC tape) measured to 1 decimal point at baseline and 90 days and/or as a composite with 90-day mortality. The key secondary outcome measures included survival to 28 and 180 days (6 months), disability-free survival to 28 days, readmission to hospital with an acute illness by 28 and 180 days, neurocognitive sequelae at 90 days (ascertained by the Developmental Milestones Checklist and an adaptation of the Kilifi Developmental Inventory^20^^,^^21^), anthropometric status at 28, 90 and 180 days (including MUAC, triceps skinfold thickness (TST) measured by the Harpenden calipers and calculated weight-for-height z-score (WHZ)) as well as adverse events associated with RUTF.

### Study methods and procedures

Eligible children were identified by a nurse and clinician on duty and registered in the eligibility screening log. All patients who met the full eligibility criteria were consented for study enrolment. Baseline clinical assessment and laboratory investigations were done at enrolment (0 h). Baseline measures of anthropometry included mid-upper arm circumference (MUAC) measured with a recommended non-stretchable tape to the nearest millimetre (supplied by UNICEF). Triceps skinfold thickness (TST) was measured at the skinfold site on the posterior surface of the arm midway between the shoulder and the elbow with a special calliper (Harpenden®) by trained research staff to the nearest 0.2 mm. Weight was measured to using electronic scales, which were regularly calibrated. Length, using a standard length board was measured for children aged <2 years old or slightly older children who were unable to stand. A stadiometer to measure height in older children used a sliding horizontal headpiece adjusted to rest on top of the head in children correctly positioned without shoes.

Two measurements were taken and the average was recorded. Research assistants at all sites were trained on taking anthropometric measurements prior to starting data collection and periodically for the duration of the study.

Children were clinically managed according to WHO guidelines. Randomisation to nutritional strategies occurred at 48 h after hospital admission/study enrolment.

### Randomisation procedure

Randomisation lists were generated by the statistician at the Intensive Care National Audit & Research Centre (ICNARC), London, and sent to the Clinical Trials Facility, KWTRP, Kilifi, Kenya. Patients were randomised using computer-generated random permuted blocks of varying sizes, stratified by trial site. An independent staff member prepared trial randomisation envelopes using these lists. The opaque envelopes contained a card with allocation and were sealed prior to distributing them to sites. The cards were numbered consecutively and opened in numerical order. Clinicians were aware of the treatment group assignments following randomisation, but the laboratory tests were run in a blinded manner.

### Treatment allocation

At 48 h following admission to hospital, eligible children were randomised (ratio 1:1) to either:

i. Supplementary feeding for 56-days (8 weeks) using one 92 g sachet (500 kcal) per day for children under 5 years or 2 sachets (1000 kcal) for children 5 years or older of RUTF in addition to their usual diet (intervention); or
ii. Usual diet alone (control, standard of care).

In stable children randomised to receive RUTF this was commenced at 48-h (after randomisation) in addition to their dietary intake. Children who were unable to tolerate oral feeds at 48-h received milk-based feeds via nasogastric tube until they were able to tolerate oral feeding. Supplemental feeds were supplied to the child during and following hospital discharge up to Day 56 post-initiation. In Kenya, the RUTF for the trial (Plumpy'nut®) was donated by UNICEF and supplied by Nutriset (France). In Uganda, the RUTF that was used in the trial (RUTAFA) was provided through the national nutrition programme. RATUFA is locally manufactured by Reco Limited (Kampala, Uganda). RUTF can be taken direct from the sachet, as it needs no preparation or dilution prior to use.

### Sample size

MUAC was selected as the primary criterion for nutritional recovery because it predicts mortality better^22^ and is also a good index of muscle mass. The primary endpoint was a composite of MUAC change from baseline (taken at admission to hospital) and mortality at 90 days. Patients were ranked firstly by their survival status at Day 90 (with death being the worst possible outcome), then by MUAC change in surviving patients, and the resulting ranks compared between arms using a two-sample rank-sum (Mann–Whitney) test with alpha of 0.05.

The original planned sample size was 2000 participants, based on anticipated recruitment to the COAST trial. Following early closure of the COAST trial in February 2020, the sample size was re-estimated using blinded data from the COAST trial to permit more accurate estimation of likely power. Based on 1645 children randomised to the COAST trial who had a mean (standard deviation) baseline MUAC of 13.7 (1.94), and assuming 80% correlation between baseline and 90-day MUAC, we expected a change in MUAC at 90 days to have a standard deviation of 1.26. Whilst there are no data on nutritional recovery in non-malnourished children, this is consistent with a recent trial publication^23^ in children with severe malnutrition (including Kilifi and Coast General Hospital, Kenya) where at 90 days, a mean change in MUAC of 1.6 cm (standard deviation: 1.1) was recorded.

We used simulations (with 10,000 simulated datasets) to calculate the sample size required to achieve 90% power. Assuming 5% loss to follow-up over both arms, and 5% 90-day mortality in the control arm, a total of 840 patients would be sufficient to detect an absolute difference in increase in MUAC of 0.3 cm together with an absolute decrease in mortality of 1%. The same sample size provided >90% power to detect a larger difference in MUAC of 0.4 cm or more together with no effect on mortality, or more than 80% power to detect a smaller difference in MUAC of 0.2 cm together with an absolute decrease in mortality of at least 3.5%.

### Study monitoring and clinical assessment

During hospital admission, all trial participants received standard of care including first and/or second parenteral antibiotics as per local or WHO guidelines). Oxygen was provided by mask or nasal canulae (for those whose SpO_2_ remained <92%) based on WHO syndromic patient management.^24^ All other care was determined by the clinical team primarily responsible for the participant's care. Children were reviewed twice daily thereafter until discharged from hospital and followed up at Day 28, Day 90 and Day 180 after the day of randomisation. At every clinical review children were actively monitored for adverse events (AE) which were reported on dedicated structured AE forms including potential relationship to randomisation arm. AEs were notified to the Clinical Trials Facility, Kilifi, Kenya and to national ethics committees within 2 days and monitored against source documents by visiting independent monitors. At each follow up, the clinician completed a symptom checklist, a targeted physical examination including anthropometry. Medical history since the last visit, including hospital readmissions (defined as an admission for acute illness which could not be managed as an outpatient), was recorded. At Day 28 post-enrolment a neurocognitive functional assessment was also conducted (using the Developmental Milestones Checklist and an adaptation of the Kilifi Developmental Inventory^20^^,^^21^). Any participant lost to follow-up before 6 months was traced for vital status (using locator data and mobile telephone contacts taken prior to discharge).

### Ethics

Ethical approval was obtained from Imperial College London Research Ethics Committee (15IC3100), School of Medicine Makerere University REC (2016–030 and the amendment 2020–155) in Uganda, and KEMRI Scientific and Ethics Review Unit (KEMRI/SERU/CGMRC-C/0053/3300 and amendment C 215/4109) in Kenya. The trial was conducted in accordance with the recommendations for research on human subjects in the Declaration of Helsinki,^25^ the ICH-GCP guidelines (E6 (R1), 1996) and the applicable national regulations.

### Statistical analysis

All clinical and laboratory data were recorded in the CRF with a unique serial number identifier. Data were entered (double data entry) onto OpenClinica Version 3 (https://www.openclinica.com/). Validation checks on the data were conducted for completeness, accuracy and consistency.

The primary outcome measure is change in MUAC from baseline to 90 days as a composite with 90-day mortality. Patients who survived to their 90-day follow-up visit were ranked by change in MUAC from baseline to 90 days. Patients who died at or before their scheduled 90-day visit were allocated the same score, which is calculated as 1 cm below the minimum recorded change in MUAC of all surviving patients (so that death is ranked as the worst possible outcome using rank-based analysis methods). Change in MUAC from baseline to 90 days, length of initial hospital stay and anthropometric endpoints (change in MUAC-for-age, skinfold thickness-for-age, weight-for-height and height-for-age z scores) at 28, 90 and 180 days were measured as continuous variables. Survival at 28, 90 and 180 days were measured as time-to-event variables. Any hospital readmission at 28 days and 180 days, neurocognitive disability at 28 days and persisting neurocognitive disability at 90 days were measured as binary variables. The primary outcome was analysed using rank-based methods (Mann–Whitney test) by intention-to-treat. The primary effect estimate was the probabilistic index—which is the probability that a randomly selected individual from the intervention arm would experience a better outcome than a randomly selected individual from the control arm. The effect size was calculated as the area under a receiver operating characteristic (ROC) curve, treating the composite outcome as a predictor of group assignment. As a sensitivity analysis, the primary outcome was also analysed using ordered logistic regression both unadjusted and adjusted for baseline MUAC, and trial site as a random effect. The individual components of the composite endpoint (change in MUAC in patients surviving to day 90, and mortality by day 90) are additionally reported by study arm, and compared using both unadjusted (t-test and log-rank test) and adjusted methods (generalised linear models and Cox regression, adjusting for baseline MUAC, and trial site as a random effect). Secondary outcomes were analysed using generalised linear models, and adjusted for baseline MUAC and trial site (as a random effect). Secondary anthropometric outcomes (change in MUAC-for-age, skinfold thickness-for-age, weight-for-height and height-for-age z scores) were adjusted for baseline measurement and trial site (as a random effect).

As the amount of missing data was anticipated to be minimal, a sensitivity approach was taken. The primary analysis was repeated under each of the following assumed scenarios:

- Patients with unknown survival at 90 days were assumed to be alive (with 0 change in MUAC) if allocated to control, or died if allocated to intervention. All patients known to be alive at 90 days but with missing MUAC had an assumed change of MUAC equal to 0.
- Patients with unknown survival at 90 days were assumed to be alive (with 0 change in MUAC) if allocated to intervention, or died if allocated to control. All patients known to be alive at 90 days but with missing MUAC had an assumed change of MUAC equal to 0.

An independent Data Monitoring Committee reviewed a single interim analysis following the recruitment and follow-up to 90 days of 385 participants, using a Haybittle-Peto stopping rule (p < 0.001) to guide recommendations to stop due to either benefit or harm.

Additional hypothesis-generating analyses investigated whether there is any evidence for a different impact of the interventions according to the following categorical variables: fever; malaria; microbiological evidence of sepsis (blood culture or retrospective molecular diagnosis); radiographic evidence of pneumonia; HIV; severe anaemia (haemoglobin <5 g/dl); and undiagnosed sickle cell disease. Subgroup analyses were conducted by testing the significance of interaction terms in the regression model for the primary outcome, as specified above.

Full details are given in the trial statistical analysis plan (additional supplemental file).

### Role of the funding source

This study is part of the EDCTP2 programme (grant number RIA-2016S-1636-COAST-Nutrition) supported by the European Union, and UK Joint Global Health Trials scheme: Medical Research Council, Department for International Development Wellcome Trust Grant Number MR/L004364/1, UK. The funding bodies did not play any role in the design of the study, data collection, data analysis & interpretation or in writing of the manuscript.

## Results

**Fig. 1:**
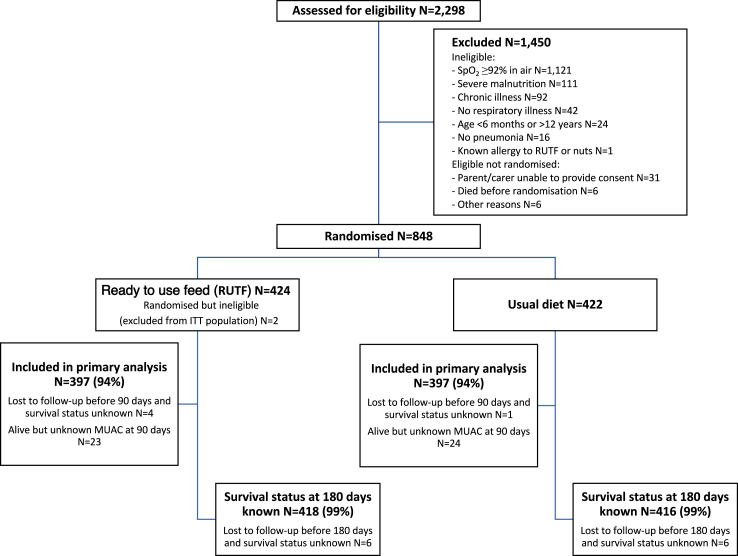
Trial profile.

Between 12 August 2018 and 22 April 2022, 2298 children were assessed for eligibility, 1450 were excluded (1407 for ineligibility; 31 declined consent; 6 died before randomisation; and 6 for other reasons) and 848 children were randomised, 426 to RUTF supplemented diet and 422 to usual diet only (control) (Fig. 1). Two children allocated to RUTF were found to be ineligible immediately post-randomisation and excluded; the remaining 846 eligible children are included in the analyses. Of those enrolled, 249 were co-enrolled in the oxygen trial, 124 randomised to RUTF supplement and 125 to usual diet (between August 2018 and February 2020), and 605 were enrolled in COAST Nutrition alone (between November 2020 and April 2022). All analyses present data for both periods combined. Baseline characteristics were balanced between randomised groups (Table 1, Table S1). Median (IQR) age was 18 months (10, 34), males were slightly more common (58%) and median (IQR) MUAC was 15 cm (14, 16), with only 27 (3%) classified as moderately malnourished (MUAC at least 11.5 cm and less than 12.5 cm). A high proportion of children had abnormal chest auscultation signs, with 637 (75%) having crepitations and 252 (30%) wheeze, and the median SpO_2_ was 89%. Radiological signs of pneumonia were present in 475 (57%) and elevated leucocyte count in 483 (58%). HIV, culture-proven bacteraemia and severe anaemia were uncommon (1%, 2% and 7%, respectively). Sickle cell disease (SCD) was reported as a working diagnosis in 41 children (5%); batch genotyping of blood stored from the admission sample showed that 101 children (12%) had SCD, which was more common in Kenyan than Ugandan children (19% vs 12%). Ceftriaxone was the most reported antibiotic (58%) (Table S2).

**Table 1:** Characteristics of children at baseline by study arm.

Parameter	Usual diet + ready to use feed (RUTF) (N = 424)	Usual diet (N = 422)	All patients (N = 846)
Age, months	17 (10, 34)	18 (10, 34)	18 (10, 34)
Male sex	244 (57.5%)	250 (59.2%)	494 (58.4%)
Female sex	180 (42.5%)	172 (40.8%)	352 (41.6%)
Mid-Upper Arm Circumference (MUAC), cm	14.8 (14.0, 15.7)	14.8 (13.8, 15.8)	14.8 (14.0, 15.8)
Undernutrition (MUAC ≥11.5 and < 12.5)	15 (3.5%)	12 (2.8%)	27 (3.2%)
Weight for age z-score	−0.7 (−1.5, 0.0)	−0.7 (−1.6, 0.1)	−0.7 (−1.5, 0.1)
Weight for height z-score	−0.4 (−1.2, 0.5)	−0.4 (−1.2, 0.5)	−0.4 (−1.2, 0.5)
Fever (>37.5 °C)	189 (44.6%)	185 (43.8%)	374 (44.2%)
History of fever	388 (91.5%)	381 (90.3%)	769 (90.9%)
History of asthma	10 (2.4%)	13 (3.1%)	23 (2.7%)
SpO_2_, %	89 (86, 90)	89 (87, 90)	89 (87, 90)
Severe hypoxaemia (SpO_2_ < 80%)	34 (8.0%)	30 (7.1%)	64 (7.6%)
Respiratory rate, per minute	55 (48, 63)	55 (48, 62)	55 (48, 62)
Tachypnoea	391 (92.2%)	380 (90.0%)	771 (91.1%)
Indrawing	385 (91.0%)	383 (90.8%)	768 (90.9%)
Cyanosis	1 (0.2%)	1 (0.2%)	2 (0.2%)
Crepitations	322 (75.9%)	315 (74.6%)	637 (75.3%)
Wheeze	122 (28.9%)	130 (30.8%)	252 (29.9%)
Pneumonia signs on chest x-ray	242 (58.0%)	233 (55.9%)	475 (57.0%)
Severe Tachycardia	108 (25.5%)	110 (26.1%)	218 (25.8%)
Compensated Shock	194 (45.8%)	191 (45.3%)	385 (45.5%)
Severe Pallor	43 (10.1%)	40 (9.5%)	83 (9.8%)
Vomiting/Diarrhoea	129 (30.5%)	127 (30.2%)	256 (30.4%)
Dehydrated	11 (2.6%)	9 (2.1%)	20 (2.4%)
Conscious level: responds to			
Pain or Voice	21 (87.5%)	25 (83.3%)	46 (85.2%)
Unresponsive	3 (12.5%)	5 (16.7%)	8 (14.8%)
Previous or recent tuberculosis diagnosis	4 (0.9%)	0 (0.0%)	4 (0.5%)
HIV	2 (0.5%)	4 (1.0%)	6 (0.7%)
Sickle Cell Disease by genotype	58 (14.2%)	43 (10.6%)	101 (12.4%)
Developmental Delay	14 (3.3%)	16 (3.8%)	30 (3.6%)
Haemoglobin, g/dl	10 (9, 12)	10 (9, 12)	10 (9, 12)
Severe Anaemia (Hb < 5 g/dl)	34 (8.1%)	27 (6.4%)	61 (7.3%)
White blood cell count, x10^3^/uL	12 (9, 18)	12 (9, 17)	12 (9, 18)
Leucocytosis (WBC>11)	245 (58.6%)	238 (56.8%)	483 (57.7%)
Malaria rapid diagnostic test	109 (26.1%)	101 (24.1%)	210 (25.1%)
Bacteraemia	10 (2.4%)	9 (2.2%)	19 (2.3%)
Lactate >2 mmol/L	148 (35.9%)	156 (37.8%)	304 (36.8%)
Antibiotics in this illness	219 (52.5%)	229 (55.4%)	448 (54.0%)
Antimalarial in this illness	116 (27.6%)	90 (21.5%)	206 (24.5%)

Data are median (interquartile range IQR) or n (%).

### Adherence

Of the 424 children randomised 4 children died in-hospital before taking the feed and one child absconded so did not receive RUTF, 419 started their RUTF supplements in hospital once they were able to take and retain feeds. The parents of 417/419 (99.5%) children randomised to RUTF supplement either carried home all the remaining sachets to cover supplementation up to Day 56 post initiation or sufficient supplies until the next clinic review at Day 28 (Table 2). At Day 28, adherence was checked by verbal enquiry to the parent/care-giver. Overall, adherence was good with 369/402 (91.8%) parents reporting RUTF was still being taken. However, only 70% reported taking all the doses and 24% took more than half the recommended doses. For the 33 children not taking RUTF at Day 28 follow-up, the most common reason was that they ran out of the sachets (72%). This resulted in a median of 7 days without taking RUTF.

**Table 2:** Adherence to allocated intervention.

Nutrition prescribed at hospital discharge	Usual diet + RUTF (N = 424)	Usual diet (N = 422)
Among patients discharged alive from hospital before 56 days:		
RUTF prescribed at discharge (any dose)	417/419 (99.5%)	0/412 (0.0%)
**Nutrition intake at 28-day follow-up visit**		
Taking RUTF at 28-day follow-up		
No	33/402 (8.2%)	406/406 (100.0%)
Yes	369/402 (91.8%)	0/406 (0.0%)
Among patients still taking RUTF at 28-day follow-up visit:		
Quantity taken		
Taking less than half recommended dose of RUTF	22/359 (6.1%)	–
Taking more than half recommended dose of RUTF	86/359 (24.0%)	–
Taking all recommended dose of RUTF	251/359 (69.9%)	–
Among patients not taking RUTF at 28-day follow-up visit:		
Reason for stopping RUTF		
Supply ran out	23/32 (71.9%)	–
Refused to take RUTF	4/32 (12.5%)	–
Other reason	5/32 (15.6%)	–
Missed days of RUTF	7 (4, 14)	
Missing	28/33 (84.8%)	–

Data are median (IQR) or n/N (%).

### Primary and secondary endpoints

**Fig. 2:**
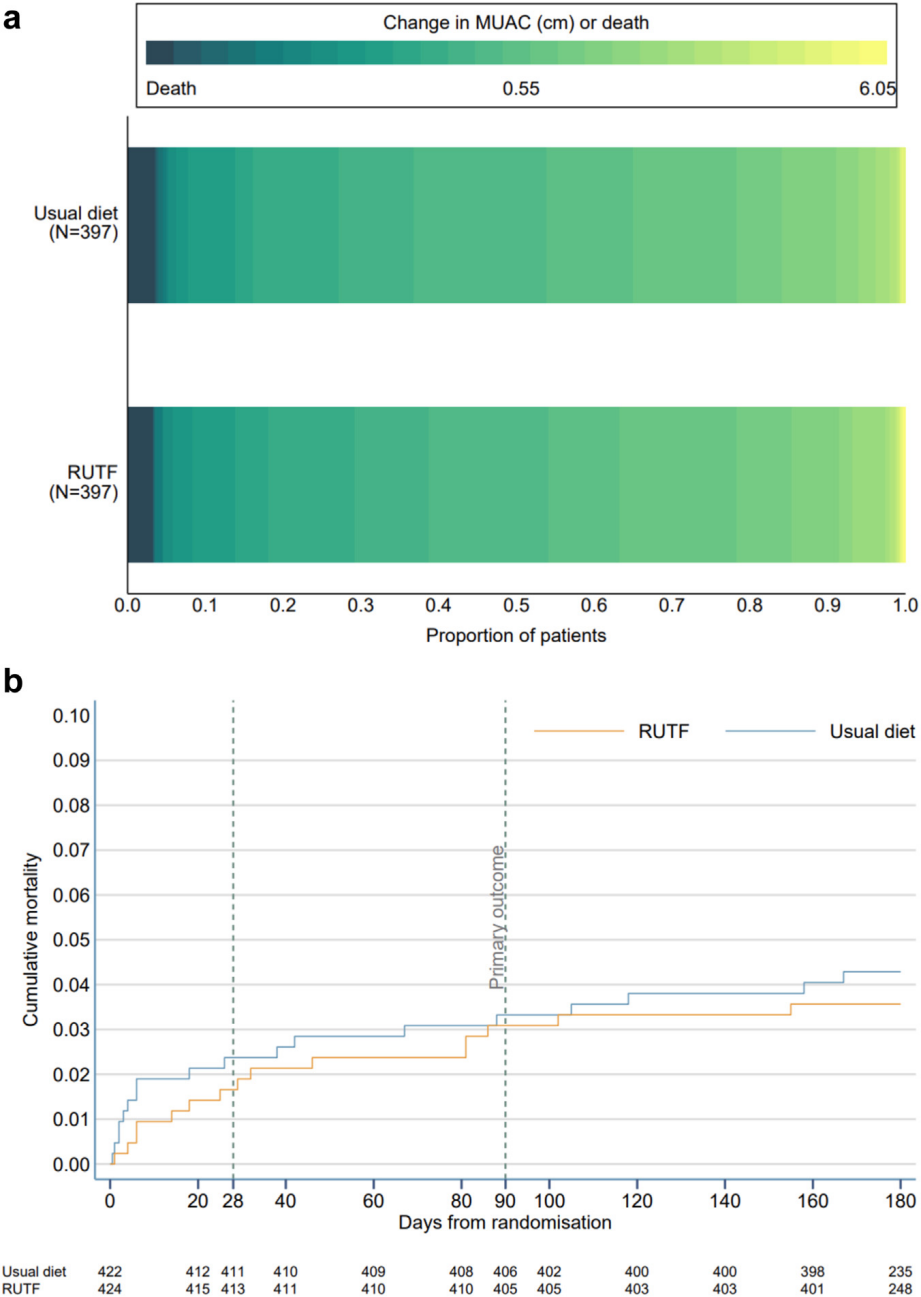
(a) Stacked bar chart illustrating composite primary outcome and (b) Kaplan–Meier plot of all-cause mortality up to day 180.

At Day 90, survival status was known for 420/424 (99.1%) and 421/422 (99.8%) and MUAC measurements were missing for 23 and 24 children in the RUTF and usual diet arms, respectively. A total of 794 participants (94%) were included in the intention to treat analysis of the primary outcome. We found no difference in the composite endpoint (probabilistic index 0.49, 95% confidence interval 0.45 to 0.53, p = 0.74) or when the separate component of the endpoint was considered (Table 3, Table S3). By Day 90, the increment in MUAC was the same (∼0.5 cm) in both arms and mortality was low with only 13/420 (3.1%) and 14/421 (3.3%) dying in the RUTF and usual diet arms, respectively. The distribution of the change in MUAC (cm) or death is compared visually as horizontally stacked proportions for each treatment group (Fig. 2a). At other time points (Days 28 and 180) there was no difference in mortality (Fig. 2b, Table 3, Table S3). Sensitivity analyses making extreme assumptions regarding missing outcome data did not alter the results (Table S4). There was no significant interaction between treatment arm and any of the pre-specified subgroups (Fig. S1).

**Table 3:** Primary and secondary outcomes.

Primary outcome	Usual diet + RUTF	Usual diet	Effect estimate (95% CI)	p value
Primary composite endpoint	0.50 (0.00, 1.00) [397]	0.50 (0.00, 1.00) [397]	0.49 (0.45, 0.53)^a^	0.740^b^
**Components of the primary outcome**				
Change in MUAC 90 d—baseline^c^	0.54 (0.85) [384]	0.55 (0.81) [383]	−0.02 (−0.13, 0.10)^d^	
Mortality by day 90	13/420 (3.1%)	14/421 (3.3%)	1.00 (0.46, 2.15)^e^	
**Secondary outcomes**				
Mortality by day 28	7/421 (1.7%)	10/421 (2.4%)	0.79 (0.29, 2.12)^e^	
Mortality by day 180	15/418 (3.6%)	18/416 (4.3%)	0.87 (0.44, 1.75)^e^	
Any hospital readmissions before 28 days^f^	17/407 (4.2%)	8/405 (2.0%)	2.2 (−0.2, 4.6)^g^	
Any hospital readmissions before 180 days^f^	54/387 (14.0%)	37/375 (9.9%)	4.1 (−0.5, 8.7)^g^	
Neurocognitive disability at 28 days^f^	7/120 (5.8%)	4/120 (3.3%)	2.5 (−2.8, 7.8)^g^	
Persisting neurocognitive disability at 90 days^f^	3/7 (42.9%)	3/4 (75.0%)	−32.1 (−88.2, 23.9)^g^	
Length of initial hospital stay, elapsed calendar days (in survivors)	3.0 (3.5) [419]	2.9 (3.0) [414]	−0.01 (−0.42, 0.41)^d^	
MUAC-for-age z-score^f^				
at 28 days	0.1 (1.1) [352]	0.0 (1.1) [350]	0.03 (−0.04, 0.11)^d^	
at 90 days	0.2 (1.1) [337]	0.2 (1.1) [330]	0.01 (−0.08, 0.10)^d^	
at 180 days	0.2 (1.1) [317]	0.2 (1.1) [297]	−0.06 (−0.17, 0.06)^d^	
Weight-for-height z-score^f^				
at 28 days	0.0 (1.3) [350]	−0.0 (1.3) [350]	0.08 (−0.04, 0.20)^d^	
at 90 days	0.2 (1.2) [336]	0.2 (1.3) [328]	−0.00 (−0.14, 0.14)^d^	
at 180 days	0.3 (1.2) [317]	0.3 (1.2) [296]	−0.03 (−0.19, 0.12)^d^	
Skinfold-for-age z-score^f^				
at 28 days	0.1 (1.1) [251]	0.0 (1.1) [246]	0.12 (0.03, 0.21)^d^	
at 90 days	0.2 (1.1) [240]	0.2 (1.1) [234]	0.14 (0.02, 0.27)^d^	
at 180 days	0.3 (1.0) [243]	0.3 (1.0) [228]	0.03 (−0.10, 0.17)^d^	

Data are median (IQR) [N], mean (SD) [N] or n/N (%) unless otherwise indicated.

a Probabilistic index.

b Wilcoxon's rank sum test.

c Calculated among patients alive and not missing MUAC at 90 days.

d Adjusted mean difference (adjusted for baseline MUAC and trial site as random effect).

e Adjusted hazard ratio (adjusted for baseline MUAC and trial site as shared frailty).

f Calculated among patients alive at follow-up timepoint.

g Absolute risk difference.

**Fig. 3:**
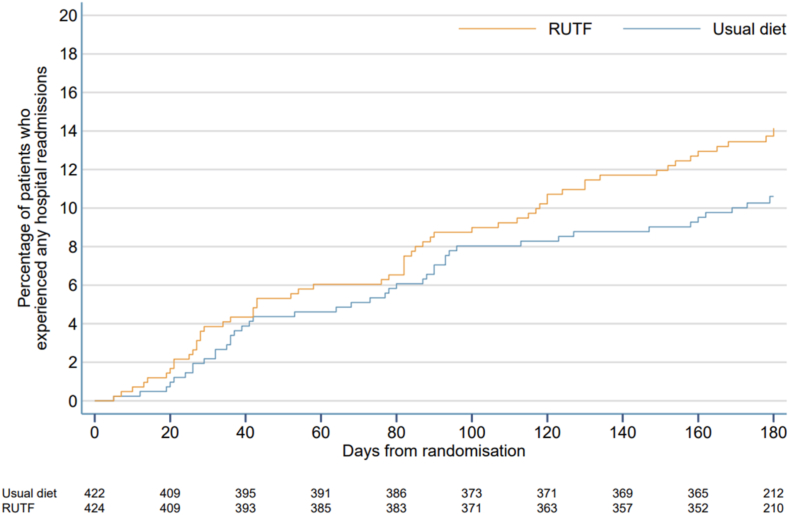
Kaplan–Meier plot of readmissions up to day 180.

Hospital readmissions were higher in the RUTF arm vs usual diet at Days 28 (4.2% vs 2.0%) and Day 180 (14.0% vs 9.9%) but this was not statistically significant when adjusted for site and baseline MUAC (odds ratio 2.34, 0.95–5.77, and 1.55, 0.98–2.45, respectively). Higher readmission rates were more common after Day 90 in the RUTF arm compared to control (Fig. 3). For all the anthropometric outcomes (change in MUAC-for-age, skinfold thickness-for-age, weight-for-height and height-for-age z scores), no differences were found at Day 90 and 180, although we found a modest improvement at Day 28 for both MUAC and weight-for-height z scores, these were not statistically significant (Table 3, Table S3, Tables S4, Fig. S2). We found greater skinfold thickness for age at Day 28 and Day 90 in the RUTF arm (Table 3, Table S3, Fig. S2) but by Day 180 there was no difference between the arms.

### Safety endpoints

Overall, more SAEs were reported in the RUTF arm (n = 164) than the usual diet arm (n = 108) (Table 4). Most adverse events reported to Day 180 occurred post-discharge from hospital and the majority were readmission with a smaller contribution of fatal events. No SAEs related to RUTF suspected allergy to RUTF and no SAEs were adjudicated as likely to be related to the intervention arm.

**Table 4:** Safety Endpoints: serious adverse events (SAEs).

Safety	Usual diet + RUTF (N = 424)	Usual diet (N = 422)
Number of patients with one or more SAEs, n (%)	69 (16.3%)	50 (11.8%)
Number of patients with a fatal SAE, n (%)	15 (3.5%)	10 (2.4%)
Total number of SAEs	164	108
Relatedness to RUTF, n (%)		
Unlikely	14 (10.8%)	–
Unrelated	98 (75.4%)	–
Not reported^a^	18 (13.8%)	–
Allergic reaction to RUTF, n/N (%)	0/414 (0.0%)	–
SAE type, number of events		
Anaemia	11	6
Cardiovascular disorders	4	8
Gastroenteritis/diarrhoea/vomiting	9	10
Hematologic disorders	5	0
Malaria	27	25
Malnutrition	1	2
Meningitis	1	0
Respiratory disorders	55	31
Septicaemia/sepsis	10	7
Sickle cell crisis	16	5
Other	25	14

a Relatedness to RUTF not reported for SAEs among children who were co-enrolled in the COAST trial.

## Discussion

We did not demonstrate any benefit in terms of mortality or anthropometric outcomes over 90 and 180 days by providing additional nutritional support using RUTF, a product which if benefit were shown could have been rapidly scaled up in public health programmes. Whilst there was no differences in MUAC (a proxy of muscle mass) we saw a modest increase in skin fold thickness (a surrogate index of body fat^26^) at Day 28 and Day 90 but no differences at Day 180. The addition of RUTF to usual diet was associated with more SAEs, specifically hospital readmissions, particularly in the period of 90–180 days follow up. The majority of admissions were for infectious-related events. The reason for this excess is unclear and may be due to chance.

One of the strengths of the trial was that for a phase 2 trial, it was a large and multisite. In terms of excellence in conduct, 359/369 (97%) followed randomised allocation before and after discharge, with none receiving RUTF in the control arm. Retention to the primary endpoint of 90 days occurred in 94% of participants. At Day 28, adherence was reasonable with 369/402 (91.8%) still taking RUTF however adherence beyond this time point was not systematically collected. It could be suggested that more strict observation that children were taking the full volume of feed may have improved adherence. This was not practical in a large trial where patients were discharged home after an average hospital stay of 2–3 days, nor would it be feasible in any future application of nutritional support within operational research or routine care.

A key limitation of the present study was the lower overall 6-month mortality (2%) than that predicted from other studies of severe pneumonia in African children, which had consistently higher mortality (∼9%). We also made a similar observation across all arms of the oxygen trial (COAST) where 28-day mortality was between 3 and 4% in the moderately severely hypoxaemic group (oxygen saturations 80–92%) but was much greater (21%) in the severely hypoxaemic group (<80%).^17^ In the COAST Nutrition trial, very few children had severe hypoxaemia, nevertheless children frequently had multiple clinical features of severe pneumonia at enrolment and >55% had an abnormal chest x-ray despite few having microbiological evidence of invasive bacterial infection. Children in the trial were overall older and better nourished than other cohorts of severe pneumonia previously reported from the continent. This may reflect the changing epidemiology of pneumonia on the continent (see below), which also impacts on the nutritional status of children presenting to hospital. The median (IQR) Paediatric Emergency triage (PET) prognostic score was 2 (1, 3) in this trial which meant children were less sick (i.e. had a better prognosis) compared to 3 (2, 4) in the control arm of the FEAST trial. This is not surprising since the baseline mortality for children with shock is greater. We had predicted a higher Day 90 mortality which was based on study data conducted prior to the widespread introduction of vaccines against the lead bacterial causes of paediatric pneumonia (*Haemophilus influenzae* type b and *Streptococcus pneumoniae*) into national immunization programs (including Uganda and Kenya) and/or the greater scaling-up of prevention and anti-retroviral medications for HIV. Over 90% of the trial participants reported up-to-date vaccination status. Which means that the range of pathogens causing severe pneumonia is likely to be similar to a more recent report of a multi-country case–control study of HIV-negative children with radiologically-confirmed pneumonia, showed that only 56/1749 (3·2%) cases had a positive blood culture. Respiratory syncytial virus, which is associated with low mortality,^27^ was the commonest pathogen.^28^

Overall, more children had serious adverse events in the RUTF arm (69, 16.3%) compared to usual diet (50, 11.8%). These mainly consisted of a higher number of readmissions. The timing of the excess readmissions did not coincide with the time when children received the RUTF (up to Day 56) with the differences between arms mainly occurring between Day 90 and Day 180. If this were due to anorectic peptides being induced by the RUTF then we would have expected to see a simultaneous decline in weight for height, MUAC or even skin-fold thickness-which were not evident.

RUTF have been designed specifically to treat the energy, protein and fat deficits of children with severe acute malnutrition. The increased metabolic requirements experience after an episode of severe pneumonia may have nutritional demands unfulfilled by RUTF. However, we are unable to comment further on this owing to the optimal baseline nutritional status of most of the children enrolled in this trial, with only three percent of children with evidence of undernutrition—the specific high risk group identified in previous studies of severe pneumonia for poor outcomes in children with undernutrition including death and readmission.^4^ The median MUAC of children in the trial was 15 cm—well above that which was used to estimate sample size (13.7 cm). This may have explained why children supplemented with RUTF tended to increase their body fat mass rather than muscle mass. Nevertheless, there are conflicting results in the relative composition of weight gain. One study, using bioimpedance to examine changes in body composition, when treated or supplemented with RUTF found ∼ 55% of weight gained was fat mass.^29^ Whereas another study in moderate malnutrition found that the main gain was fat-free tissue when rehabilitated with lipid-based nutrient or corn-soy blend feeds.^30^ Whether this predisposed children to increased morbidity (readmissions) is unknown.

In conclusion, future trials of nutritional support following pneumonia should aim to focus on the high risk undernourished group and use feeds designed to target the metabolic needs of children with severe infection to optimise outcomes. Additional considerations for the composition of nutritional supplementation should also include safety and efficacy in the acute and recovery phases of illnesses.

## Contributors

KM, AB, KR, DAH, POO and SK designed the study; SK POO, MH, FA, ROO, AT, SO, DN, EN, WO, MN, DA, RM, TNW gathered the data; AM, HM and EO were responsibility for project administration; EG, KT and DAH accessed and analyzed the data and can vouch for the underlying data; EG prepared the figures. All authors had full access to all the data in the study. All authors accept responsibility for the decision to submit for publication. KM wrote the original draft paper which all authors commented on.

## Data sharing statement

The datasets used and/or analysed during the current study are available from the corresponding author on reasonable request from one year after publication (k.maitland@imperial.ac.uk) following approval, with signed data access agreement, of a proposal by the Trial Management Team and if necessary the Trial Steering Committee. This period of one year enables the COAST team to prepare manuscripts of other planned analyses.

Data available will be partially de-identified and will have a linked data dictionary.

## Declaration of interests

The authors declare that they have no competing interests.

## Appendix A Supplementary data

Supplementary Tables and Figures

COAST Nutrition

COAST Nutrition SAP v1

## References

[1] Black R.E., Cousens S., Johnson H.L. (2010). Global, regional, and national causes of child mortality in 2008: a systematic analysis. Lancet.

[2] Moisi J.C., Gatakaa H., Berkley J.A. (2011). Excess child mortality after discharge from hospital in Kilifi, Kenya: a retrospective cohort analysis. Bull World Health Organ.

[3] Banajeh S.M. (1998). Outcome for children under 5 years hospitalized with severe acute lower respiratory tract infections in Yemen: a 5 year experience. J Trop Pediatr.

[4] Ngari M.M., Fegan G., Mwangome M.K. (2017). Mortality after inpatient treatment for severe pneumonia in children: a cohort study. Paediatr Perinat Epidemiol.

[5] Qazi S., Aboubaker S., MacLean R. (2015). Ending preventable child deaths from pneumonia and diarrhoea by 2025. Development of the integrated global action plan for the prevention and control of pneumonia and diarrhoea. Arch Dis Child.

[6] Baudouin S.V., Evans T.W. (2003). Nutritional support in critical care. Clin Chest Med.

[7] Debaveye Y., Van den Berghe G. (2006). Risks and benefits of nutritional support during critical illness. Annu Rev Nutr.

[8] Reeds P.J., Fjeld C.R., Jahoor F. (1994). Do the differences between the amino acid compositions of acute-phase and muscle proteins have a bearing on nitrogen loss in traumatic states?. J Nutr.

[9] Ziegler T.R., Gatzen C., Wilmore D.W. (1994). Strategies for attenuating protein-catabolic responses in the critically ill. Annu Rev Med.

[10] Shoham J., Duffield A. (2009). Proceedings of the World health organization/UNICEF/World food programme/united Nations high commissioner for refugees consultation on the management of moderate malnutrition in children under 5 years of age. Food Nutr Bull.

[11] Golden M.H. (2009). Proposed recommended nutrient densities for moderately malnourished children. Food Nutr Bull.

[12] (2022). Guidelines for ready-to-use therapeutic foods (RUTF) CXG 95-2022.

[13] Bailey J., Chase R., Kerac M. (2016).

[14] Maust A., Koroma A.S., Abla C. (2015). Severe and moderate acute malnutrition can Be successfully managed with an integrated protocol in sierra leone. J Nutr.

[15] Briend A., Khara T., Dolan C. (2015). Wasting and stunting--similarities and differences: policy and programmatic implications. Food Nutr Bull.

[16] Kiguli S., Olopot-Olupot P., Alaroker F. (2021). Children's oxygen administration strategies and nutrition trial (COAST-Nutrition): a protocol for a phase II randomised controlled trial. Wellcome Open Res.

[17] Maitland K., Kiguli S., Olupot-Olupot P. (2021). Randomised controlled trial of oxygen therapy and high-flow nasal therapy in African children with pneumonia. Intensive Care Med.

[18] (2013). Pocket book of hospital care for children: guidelines for the management of common childhood illnesses.

[19] Maitland K., Molyneux S., Boga M., Kiguli S., Lang T. (2011). Use of deferred consent for severely ill children in a multi-centre phase III trial. Trials.

[20] Abubakar A., Holding P., Van de Vijver F., Bomu G., Van Baar A. (2010). Developmental monitoring using caregiver reports in a resource-limited setting: the case of Kilifi, Kenya. Acta Paediatr.

[21] Abubakar A., Holding P., van Baar A., Newton C.R., van de Vijver F.J. (2008). Monitoring psychomotor development in a resource-limited setting: an evaluation of the Kilifi Developmental Inventory. Ann Trop Paediatr.

[22] Bejon P., Mohammed S., Mwangi I. (2008). Fraction of all hospital admissions and deaths attributable to malnutrition among children in rural Kenya. Am J Clin Nutr.

[23] Berkley J.A., Ngari M., Thitiri J. (2016). Daily co-trimoxazole prophylaxis to prevent mortality in children with complicated severe acute malnutrition: a multicentre, double-blind, randomised placebo-controlled trial. Lancet Glob Health.

[24] Hospital Care for Children (2013).

[25] (1964). Declaration of Helsinki - ethical principles for medical research involving human subjects.

[26] Davis M.C. (2007). Handbook of physiological research methods in Health Psychol.

[27] Nokes D.J., Ngama M., Bett A. (2009). Incidence and severity of respiratory syncytial virus pneumonia in rural Kenyan children identified through hospital surveillance. Clin Infect Dis.

[28] Pneumonia Etiology Research for Child Health Study G (2019). Causes of severe pneumonia requiring hospital admission in children without HIV infection from Africa and Asia: the PERCH multi-country case-control study. Lancet.

[29] Kangas S.T., Kaestel P., Salpeteur C. (2020). Body composition during outpatient treatment of severe acute malnutrition: results from a randomised trial testing different doses of ready-to-use therapeutic foods. Clin Nutr.

[30] ENN ENN (2018). Effectiveness of food supplements in increasing fat-free tissue accretion in children with moderate acute malnutrition in Burkina Faso. Field Exchange.

