# Supplementary material for: Nutritional supplementation in children with severe pneumonia in Uganda and Kenya (COAST-Nutrition): a phase 2 randomised controlled trial
Source: eClinicalMedicine. 2024 May 14;72:102640. doi: 10.1016/j.eclinm.2024.102640 (PMC11106534; doi:10.1016/j.eclinm.2024.102640)

Supplementary Methods Tables and Figures

Table of Contents

JUSTIFICATION FOR OXYGEN ADMINISTRATION THRESHOLD < 92%. **2**

TABLE S1. ADDITIONAL BASELINE CHARACTERISTICS AND WORKING DIAGNOSIS. **3**

TABLE S2. ANTIBIOTICS GIVEN TO CHILDREN DURING ADMISSION. **5**

TABLE S3. ADDITIONAL ANALYSES OF PRIMARY AND SECONDARY OUTCOMES. **6**

TABLE S4 PRIMARY OUTCOME SENSITIVITY ANALYSES **7**

TABLE S5. ADDITIONAL SECONDARY OUTCOMES .... ERROR! BOOKMARK NOT DEFINED.

FIGURE S1 SUBGROUP ANALYSES OF THE PRIMARY COMPOSITE OUTCOME. **8**

FIGURE S2 CHANGES IN MEDIAN (A) MUAC (CM), (B) TRICEPS SKINFOLD THICKNESS (MM) AND (C) WEIGHT-FOR-HEIGHT Z-SCORE AT 28, 90 AND 180 DAYS. **9**

Justification for oxygen administration threshold < 92%.

The lack of evidence that maintaining 'normal' oxygen values in critically ill patients is beneficial possibly explains the wide variation in practice surrounding the management of hypoxia. For children in resource-poor countries, the 2013 WHO recommended threshold for giving oxygen therapy is SpO_2 <90%, or SpO_2 <87% if living at altitude >2500m [9]. This was graded as a strong recommendation based on low quality of evidence. Whilst Oxygen is recommended for those with oxygen saturations of <90%, most hospitals in Africa lack the facility to measure this. Therefore the guidelines recommend, where clinicians do not have access to pulse oximetry, the following the clinical signs to initiate oxygen therapy: a child with a history of cough and difficulty one or more of signs of putative pneumonia (respiratory distress, evidence of central cyanosis) or the presence of danger signs (lethargy or greater, convulsions).

In the Childrens Oxygen Administration Strategies Trial (COAST) we selected a higher threshold for investigation (<92% rather than <90%) enables the trial to investigate whether, if oxygen therapy is beneficial, the WHO threshold should be revised to include targeting a higher threshold (which is compatible with international recommendations). Logically, following on from this argument, if we had targeted < 90% and the COAST trial confirms that oxygen is life-saving then a future trial to examine whether it should be targeted to children with saturations 90-91% would require a sample size of substantial proportions, with little hope of securing funding. The COAST trial includes a health economic evaluation (from a health services perspective) so we will be able to provide future policy makers with data on both the clinical and cost-effectiveness of alternative oxygen strategies, taking into account baseline oxygen saturation. Our trial was endorsed ISARIC at a meeting in Annecy, July 2012. The meeting brought together many leading researchers and clinical trialists in severe respiratory illnesses with representation from international agencies, such as the WHO. The proposal had wide support as it was anticipated that the trial would result in refinements to the current WHO recommendations and provide a robust evidence to support the targeting of oxygen to those with the greatest need thereby averting unnecessary consumption in financially constrained hospitals. The pragmatic nature of COAST and locations chosen will mean that the results of the trial will be immediately generalisable.

Table S1. Additional baseline characteristics and working diagnosis.

Parameter	Usual diet+RUTF (N=424)	Usual diet (N=422)	All patients (N=846)
Respiratory Severity Distress score			
0	251 (59.2%)	229 (54.3%)	480 (56.7%)
1-3	140 (33.0%)	162 (38.4%)	302 (35.7%)
4-6	31 (7.3%)	30 (7.1%)	61 (7.2%)
7-9	2 (0.5%)	1 (0.2%)	3 (0.4%)
Ability to feed			
Normal	307 (72.4%)	298 (70.6%)	605 (71.5%)
Some difficulty	96 (22.6%)	105 (24.9%)	201 (23.8%)
Severe difficulty	14 (3.3%)	8 (1.9%)	22 (2.6%)
Unable	7 (1.7%)	11 (2.6%)	18 (2.1%)
Altered consciousness	29 (6.9%)	29 (6.9%)	58 (6.9%)
Fits in the illness	27 (6.4%)	23 (5.5%)	50 (5.9%)
Neck stiffness/bulging font	4 (1.0%)	3 (0.7%)	7 (0.8%)
Prior admission to another facility for > 24 hours	37 (8.7%)	32 (7.6%)	69 (8.2%)
Vomiting	114 (26.9%)	100 (23.7%)	214 (25.3%)
Diarrhoea > 3 loose stools/day	44 (10.4%)	50 (11.8%)	94 (11.1%)
**Past history**			
Gestational age < 37 weeks*	19 (4.5%)	19 (4.5%)	38 (4.5%)
Breast fed exclusively for at least 3 months:	389 (92.0%)	386 (91.7%)	775 (91.8%)
Admitted to hospital in first month of life	53 (12.5%)	41 (9.7%)	94 (11.1%)
Two or more hospital admissions this year	56 (13.2%)	69 (16.4%)	125 (14.8%)
Known epilepsy	0 (0.0%)	1 (0.2%)	1 (0.1%)
Vaccination status up to date	272 (90.4%)	269 (90.6%)	541 (90.5%)
**Laboratory parameters**			
Hypoglycaemia (glucose <3 mmol/L)	15 (3.6%)	16 (3.8%)	31 (3.7%)
Sodium, mmol/L	135 (131, 138)	134 (131, 138)	135 (131, 138)
Missing sodium values	387 (91.3%)	385 (91.2%)	772 (91.3%)
Potassium, mmol/L	4 (4, 5)	4 (4, 4)	4 (4, 5)
Missing potassium values	387 (91.3%)	385 (91.2%)	772 (91.3%)
Creatinine, mmol/L	40 (24, 49)	42 (17, 49)	42 (24, 49)
Missing creatinine values	389 (91.7%)	385 (91.2%)	774 (91.5%)
Urea, umol/L	2 (1, 4)	4 (2, 6)	3 (2, 5)
Missing urea values	394 (92.9%)	387 (91.7%)	781 (92.3%)
Malaria Slide positive* n %	33 (7.9)	40 (9.5)	73 (8.7)
FEAST Paediatric Emergency Triage (PET) score			
0 (%)	8 (1.9)	8 (1.9)	16 (1.9)
1 (%)	113 (26.7)	111 (26.3)	224 (26.5)
2 (%)	196 (46.2)	176 (41.7)	372 (44.0)
3 (%)	76 (17.9)	88 (20.9)	164 (19.4)
4 (%)	16 (3.8)	29 (6.9)	45 (5.3)
5+ (%)	15 (3.5)	10 (2.4)	25 (3.0)
Median (IQR)	2 (1, 3)	2 (1, 3)	2 (1, 3)

Continued

**Working diagnoses**			
LRTI – any	407 (96.0%)	401 (95.0%)	808 (95.5%)
Tuberculosis	5 (1.2%)	6 (1.4%)	11 (1.3%)
Severe malaria – all forms	78 (18.4%)	80 (19.0%)	158 (18.7%)
Sepsis/septicaemia	26 (6.1%)	24 (5.7%)	50 (5.9%)
Severe anaemia	37 (8.7%)	30 (7.1%)	67 (7.9%)
Sickle cell disease	24 (5.7%)	17 (4.0%)	41 (4.8%)
Malnutrition – any	5 (1.2%)	5 (1.2%)	10 (1.2%)
Asthma	22 (5.2%)	31 (7.3%)	53 (6.3%)
Gastroenteritis/diarrhoea	8 (1.9%)	13 (3.1%)	21 (2.5%)
URTI – any	12 (2.8%)	17 (4.0%)	29 (3.4%)
Developmental delay/cerebral palsy	1 (0.2%)	4 (0.9%)	5 (0.6%)
Heart condition, congenital or other	3 (0.7%)	0 (0.0%)	3 (0.4%)
Bronchiolitis	6 (1.4%)	3 (0.7%)	9 (1.1%)
Measles	4 (0.9%)	1 (0.2%)	5 (0.6%)
HIV/AIDS	2 (0.5%)	5 (1.2%)	7 (0.8%)
Meningitis or encephalitis	7 (1.7%)	9 (2.1%)	16 (1.9%)
Other chest diagnosis	11 (2.6%)	3 (0.7%)	14 (1.7%)

Data reported are medians (IQR) or n (%)

Table S2. Antibiotics given to children during admission.

Antibiotics	Usual diet+RUTF (N=103)	Usual diet (N=108)	All patients (N=211)
Benzylpenicillin	6 (5.8%)	4 (3.7%)	10 (4.7%)
Ampicillin	10 (9.7%)	12 (11.1%)	22 (10.4%)
Gentamicin	11 (10.7%)	13 (12.0%)	24 (11.4%)
Penicillin IV	7 (6.8%)	11 (10.2%)	18 (8.5%)
Hydrocortisone	1 (1.0%)	2 (1.9%)	3 (1.4%)
Ceftriaxone	62 (60.2%)	61 (56.5%)	123 (58.3%)
Chloramphenicol	1 (1.0%)	0 (0.0%)	1 (0.5%)
Cefotaxime	0 (0.0%)	2 (1.9%)	2 (0.9%)
Metronidazole	1 (1.0%)	1 (0.9%)	2 (0.9%)
Artesunate	1 (1.0%)	0 (0.0%)	1 (0.5%)
Cloxacillin	1 (1.0%)	0 (0.0%)	1 (0.5%)
Flucox-amoxillicin	0 (0.0%)	1 (0.9%)	1 (0.5%)
Unknown	2 (1.9%)	1 (0.9%)	3 (1.4%)

Data are n (%)

Table S3. Additional analyses of primary and secondary outcomes

**Primary outcome**	**Effect estimate (95% CI)**
Primary composite endpoint	0.96 (0.75, 1.22) [a]
	0.95 (0.74, 1.20) [b]
**Components of the primary outcome**	
Change in MUAC 90d – baseline [c]	-0.01 (-0.13, 0.11) [d]
Mortality by day 90	0.99 (0.46, 2.14) [e]
**Secondary outcomes**	
Mortality by day 28	0.77 (0.29, 2.07) [e]
Mortality by day 180	0.88 (0.44, 1.76) [e]
Any hospital readmissions before 28 days [f]	2.16 (0.92, 5.07) [a]
	2.17 (0.92, 5.11) [b]
Any hospital readmissions before 180 days [f]	1.48 (0.95, 2.31) [a]
	1.55 (0.98, 2.45) [b]
Neurocognitive disability at 28 days [f]	1.80 (0.51, 6.30) [a]
	1.92 (0.49, 7.45) [b]
Persisting neurocognitive disability at 90 days [f]	0.25 (0.02, 3.77) [a]
	0.19 (0.01, 3.78) [b]
Length of initial hospital stay, elapsed calendar days (in survivors)	0.02 (-0.42, 0.46) [d]
MUAC-for-age z-score [f]	
at 28 days	0.06 (-0.11, 0.22) [d]
at 90 days	0.03 (-0.14, 0.20) [d]
at 180 days	-0.05 (-0.23, 0.12) [d]
Weight-for-height z-score [f]	
at 28 days	0.07 (-0.12, 0.27) [d]
at 90 days	-0.00 (-0.19, 0.19) [d]
at 180 days	-0.08 (-0.27, 0.11) [d]
Skinfold-for-age z-score [f]	
at 28 days	0.03 (-0.16, 0.22) [d]
at 90 days	0.07 (-0.13, 0.26) [d]
at 180 days	-0.03 (-0.22, 0.15) [d]
Height-for-age z-score [f]	
at 28 days	-0.01 (-0.22, 0.20) [d]
at 90 days	0.09 (-0.12, 0.31) [d]
at 180 days	0.06 (-0.15, 0.26) [d]

[a] Unadjusted odds ratio; [b] Adjusted odds ratio (adjusted for baseline MUAC and trial site as random effect); [c] Calculated among patients alive and not missing MUAC at 90 days; [d] Unadjusted mean difference; [e] Unadjusted hazard ratio; [f] Calculated among patients alive at follow-up timepoint.

Table S4 Primary outcome sensitivity analyses.

Sensitivity analysis of the primary outcome	Usual diet+RUTF	Usual diet	Effect estimate (95% CI)
**Worst case scenario**			
Primary composite endpoint, median (IQR) [N]	0.40 (0.00, 1.00) [424]	0.50 (0.00, 1.00) [422]	0.49 (0.45, 0.53) [a]
			0.94 (0.75, 1.19) [b]
			0.93 (0.74, 1.18) [c]
**Best case scenario**			
Primary composite endpoint, median (IQR) [N]	0.40 (0.00, 1.00) [424]	0.50 (0.00, 1.00) [422]	0.49 (0.45, 0.53) [a]
			0.96 (0.76, 1.21) [b]
			0.95 (0.75, 1.20) [c]

[a] Probabilistic index; [b] Unadjusted odds ratio; [c] Adjusted odds ratio (adjusted for baseline MUAC, and trial site as random effect).

Figure S1 Subgroup analyses of the primary composite outcome.

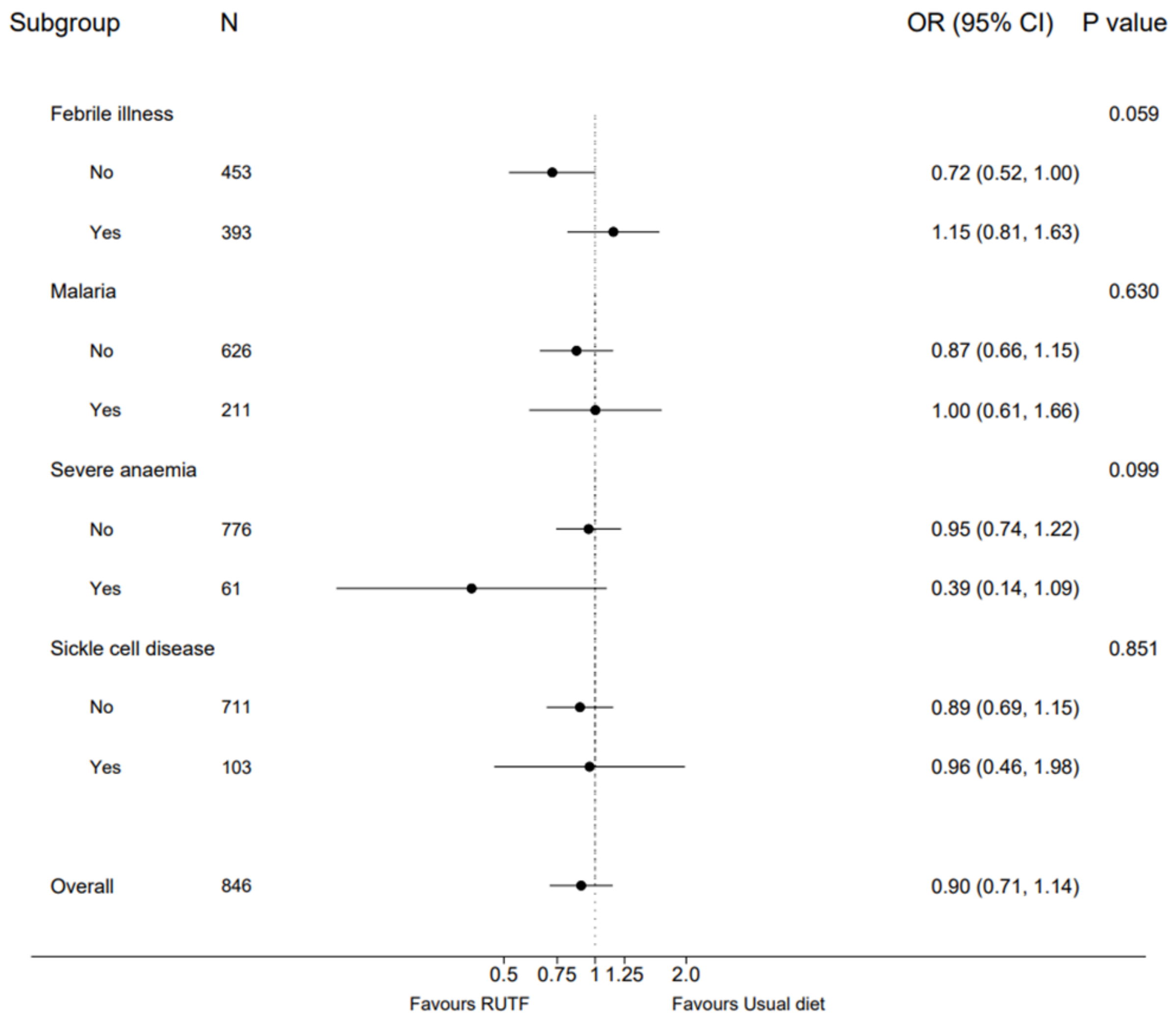

Figure S2 Changes in median (a) MUAC (cm), (b) triceps skinfold thickness (mm) and (c) weight-for-height z-score at 28, 90 and 180 days.

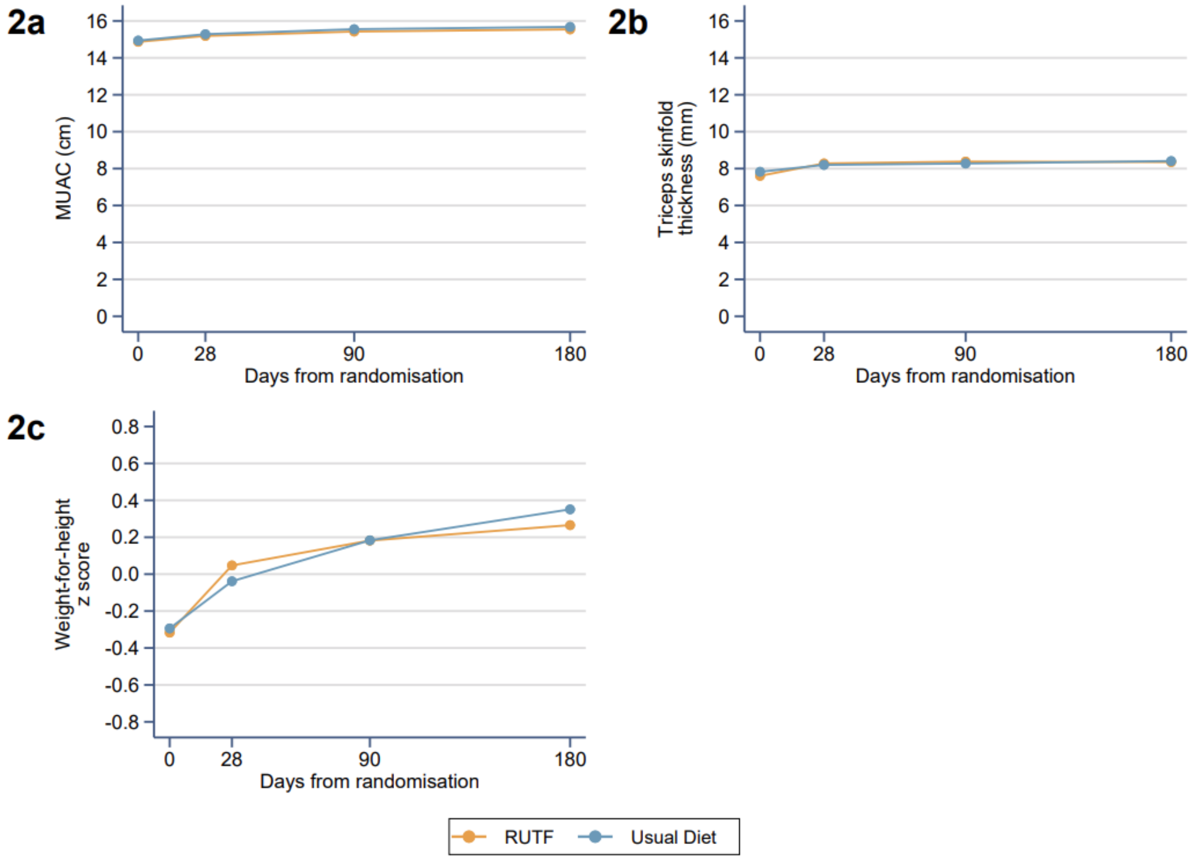

Supplement: Supplementary Tables and Figures [file mmc1.pdf]
