# Supplementary material for: Nutritional supplementation in children with severe pneumonia in Uganda and Kenya (COAST-Nutrition): a phase 2 randomised controlled trial
Source: eClinicalMedicine. 2024 May 14;72:102640. doi: 10.1016/j.eclinm.2024.102640 (PMC11106534; doi:10.1016/j.eclinm.2024.102640)
Supplement: COAST Nutrition SAP v1 [file mmc3.pdf]

COAST-Nutrition

Statistical Analysis Plan

Version 1.0

**Protocol version & date**	4.0 17th February 2020
**ICREC number:**	15IC3100
**Trial sponsor:**	Imperial College London
**Trial sponsor reference:**	P46493
**Trial funder(s):**	1/ Joint Global Health Trials scheme:<br>Medical Research Council Department for International Development Wellcome Trust<br>2/ European and Developing Countries Clinical Trials Partnership (EDCTP)
**Funder reference:**	MR/L004364/1<br>RIA2016S-1636
**ISRCTN number:**	ISRCTN10829073
**Chief investigator:**	Prof Kathryn Maitland
**Author:**	Karen Thomas

Roles, responsibility, signatures

Role	Name	Signature	Date
Chief Investigator	Prof Kathryn Maitland		25th May 2022
Head Statistician	Prof David Harrison		11th July 2022
Author	Ms Karen Thomas		11th July 2022

Version history

Version number	Date	Summary of main changes from previous versions	Timing
1.0		N/A	

Contents

Abbreviations 4

1. Background 5

2. Objective and hypotheses 5

3. Study design 5

 3.1 Inclusion/exclusion criteria 5

  3.1.1 Inclusion criteria 5

  3.1.2 Exclusion criteria 6

 3.2 Study Treatments 6

 3.3 Randomisation 6

 3.4 Outcome measures 6

  3.4.1 Primary 6

  3.4.2 Secondary 6

 3.5 Framework 7

 3.6 Timing of final analysis 7

 3.7 Timing of outcome assessments 7

 3.8 Economic analysis and further substudies 8

4. Sample size calculation 8

5. End of trial 8

6. Analysis principles 8

7. Handling of missing data 9

8. Analysis populations 9

9. Initial descriptive analyses 9

 9.1 Recruitment, treatment allocation and follow-up 9

 9.2 Baseline characteristics 10

 9.3 Protocol adherence 13

 9.4 Withdrawals 13

10. Primary analysis 13

11. Secondary analyses 14

12. Subgroup analyses 14

13. Interim analyses 15

14. Timing of primary analysis 15

15. Safety 15

16. Statistical software 15

References 16

Abbreviations

COAST	Children's Oxygen Administration Strategies Trial
DMEC	Data monitoring and ethics committee
ERC	Endpoint review committee
FiO_2	Oxygen concentration
ICNARC	Intensive Care National Audit & Research Centre
IQR	interquartile range
ITT	Intention to treat
mITT	Modified intention to treat
LRTI	lower respiratory tract infection
MUAC	Mid upper arm circumference
RUTF	Ready to use feed
SAE	Serious adverse event
SBP	Systolic blood pressure
SD	Standard deviation
SpO_2	Oxygen saturation
TST	Triceps skinfold thickness
WBC	White blood count
WHO	World Health Organization
URTI	Upper respiratory tract infection
VSP	Very severe pneumonia

1. Background

The COAST-Nutrition trial is a open, multicentre, randomised controlled trial of supplementary feeding vs. usual care in children admitted to hospital with respiratory distress complicated by hypoxia (SpO_2<92%)

This document describes the proposed statistical analyses for the primary and secondary clinical endpoints of the Trial. It is important to set these out and agree on them in advance of analysing the outcome data for the Trial, so that data-derived decisions in the analyses are avoided. This statistical analysis plan has been prepared in accordance with recent published guidelines[1].

2. Objective and hypotheses

The primary objective of the Trial is to determine whether supplementary feeding for 56-days (8 weeks) using Ready to Use Therapeutic Feeds (RUTF) in addition to usual diet in children aged 6 months or more and without severe acute malnutrition versus usual standard care (usual diet alone) will improve outcome (mid-upper arm circumference and mortality) at 90-days.

3. Study design

3.1 Inclusion/exclusion criteria

The Trial population will consist of infants and children receiving treatment at a participating site who fulfil all of the inclusion criteria and none of the exclusion criteria below.

3.1.1 Inclusion criteria

- Aged between 6 months to 12 years
- History of respiratory illness (cough, upper respiratory tract symptom or any respiratory symptoms, e.g. rapid breathing or increase work of breathing)
- Hypoxia (pulse oximetry reading of SpO_2 <92% recorded in room air over 5 minutes) Plus suspected severe pneumonia informed by WHO guidelines, as below –
  a. Sign of respiratory distress (any one of):
     - severe lower chest wall in-drawing
     - use of auxiliary muscles
     - head nodding
     - inability to feed because of respiratory problems
  b. Suspected pneumonia
     - fast breathing:
       - age 6–11 months: ≥ 50/minute
       - age 1–5 years: ≥ 40/minute
       - age 5-12 years ≥ 30/minute
     - chest auscultation signs:
       - decreased breath sounds
       - bronchial breath sounds
       - crackles

- abnormal vocal resonance (decreased over a pleural effusion or empyema, increased over lobar consolidation)
- pleural rub

c. Signs of pneumonia with a general danger sign:
   - inability to breastfeed or drink
   - lethargy or unconscious
   - convulsions

3.1.2 Exclusion criteria

- Severe malnutrition (MUAC <11.5cm)
- Consent refusal by parent/carer
- Previously recruited to COAST-Nutrition

3.2 Study Treatments

Children randomised to receive RUTF will receive one 92 g sachet (500 Kcal) per day of Ready to Use Therapeutic Feeds (RUTF) in addition to their dietary intake, commencing after randomisation at 48-hours. Children unable to tolerate oral feeds at 48-hours will receive milk-based feeds via nasogastric tube until they are able to tolerate oral feeding. Children randomised to usual care will remain on their usual diet. All other care will be determined by the clinical team primarily responsible for the participant's care, as per standard clinical practise.

3.3 Randomisation

Patients will be screened at hospital admission. For eligible patients, consent will be sought from parents/guardians and baseline data will be collected on the first day of admission. Patients who remain eligible for the trial will then be randomised at 48 hours following hospital admission, using computer-generated random permuted blocks of varying sizes, stratified by trial site.

3.4 Outcome measures

3.4.1 Primary

The primary outcome measure is change in mid-upper arm circumference (MUAC) from baseline to 90 days as a composite with 90-day mortality. Patients who survive to their 90-days follow-up visit will be ranked by change in MUAC from baseline to 90 days. Patients who have died at or before their scheduled 90-day visit will all be allocated the same score, which is calculated as equal to 1cm below the minimum recorded change in MUAC of all surviving patients (so that death is ranked as the worst possible outcome using rank-based analysis methods).

3.4.2 Secondary

The secondary outcome measures are:

- Survival to 28 days and 180 days (6 months), which is measured using time to event methods, with surviving patients censored at date of last known follow-up

- Disability-free survival to 28 days, with patients classified as alive with no disability if they were alive at their scheduled 28-day visit with none of the following conditions recorded: Abnormal vision; abnormal hearing; abnormal speech production; abnormal comprehension; abnormal behaviour; abnormal movement/motor function; feeding difficulty; fail on gross motor or fine motor function.

- Re-admission to hospital by 28 and 180 days, which will be measured as a binary outcome, with patients who have recorded any re-admission by the time of their 28 day (or 180 day) visit (or who have remained in hospital from randomisation to day 28 (day 180) including as having been re-admitted, and all patients who and were discharged from their initial hospital stay before 28 days (180 days) and who survive to their 28 day (180 day) visit classified as not being readmitted.

- Resolution of neurocognitive sequelae at 90 days, measured in all children who have any neurological deficit recorded at 28 days and who have been assessed at 90 days, with the 28 day neurological deficit classed as resolved by 90 days only of no deficit of any kind (new or continued) is recorded at 90 days. A neurological deficit is defined as the presence of any one or more of the following: abnormal speech production; abnormal comprehension; abnormal behaviour; abnormal movement/motor function; feeding difficulty; fail on gross motor or fine motor function.

- MUAC, triceps skinfold thickness (TST), weight and height by 28, 90 and 180 days. MUAC and TST are measured in cm at 28 and 90 days. Weight in kg and height in cm is measured at 28 and 90 days, and each will be converted to age-corrected z-scores using the Stata macro igrowup_standard.ado published by the World Health Organization.

- Frequency of any adverse events associated with RUTF, measured as the number and percentage of patients with any allergic reaction to RUTF recorded at any follow-up date, and as the number and percentage of patients with any SAE assessed as definitely, probably or possibly related to RUTF (SAEs are reported up to 180 days following randomization).

3.5 Framework

All statistical tests will be for superiority.

3.6 Timing of final analysis

The final analysis will be performed only after all patients recruited to the trial have completed their 180 day follow-up.

3.7 Timing of outcome assessments

All participants will be followed up either in hospital (if not yet discharged) or in person at a clinic visit at 28, 90 and 180 days post randomisation. Follow-up visits should be scheduled as close as possible to the specified day, but there is no absolute restriction placed on the allowed visit windows.

3.8 Economic analysis and further substudies

In additional to the primary and secondary clinical endpoints covered by this plan, there are a number of other ancillary studies planned including (but not limited to) a comparison of cost-effectiveness of the intervention compared to normal care, investigations of various biomarkers (including molecular diagnostics, and anthropometric measures) and their association with mortality. All these analyses will be performed and reported separately to the main clinical endpoints of the trial, and are not further described in this SAP.

4. Sample size calculation

The primary endpoint is a composite of MUAC change from baseline to day 90, and mortality at 90 days. Patients will be ranked firstly by their survival status at day 90 (with death being the worst possible outcome), then by MUAC change in surviving patients, and the resulting ranks will be compared between arms using a two-sample rank-sum (Mann-Whitney) test with alpha of 0.05.

Based on 1,645 children randomised to the COAST trial who had a mean (sd) baseline MUAC of 13.7 (1.94), and assuming 80% correlation between baseline and 90 day MUAC, we can expect change in MUAC scores at 90 days to have a standard deviation of 1.26.

We used simulations (with 10,000 simulated datasets) to calculate the sample size required to achieve 90% power. Assuming 5% loss to follow-up over both arms, and 5% mortality by 90 days in the control arm, a total of 840 patients would be sufficient to detect an absolute increase in MUAC change of 0.3cm together with an absolute decrease in mortality of 1%. The same sample size also provides more than 90% power to detect a larger difference in MUAC of 0.4cm or more together with no effect on mortality, or more than 80% power to detect a smaller difference in MUAC of 0.2cm together with an absolute decrease in mortality of at least 3.5%.

5. End of trial

The end of the trial will be when the final participant has completed their 180-day follow-up.

6. Analysis principles

All analyses will be conducted by intention to treat. The infants and children will be analysed according to the treatment group they were randomised to, irrespective of whether the treatment allocated was received, i.e. all infants and children will be included in the analysis, regardless of whether they have, or have not, adhered to the protocol. A two-sided p value of <0.05 will be taken to indicate a statistically significant result. No correction for multiple testing will be made. Effect estimates will be reported with 95% confidence intervals.

7. Handling of missing data

As the amount of missing data is anticipated to be minimal, a sensitivity approach will be taken when the primary outcome variable is missing. In the event that there are any missing outcomes at 90 days, the primary analysis will be repeated under each of the following assumed scenarios:

> Patients with unknown survival at 90 days will be assumed to be alive (with 0 change in MUAC) if allocated to control, or died if allocated to intervention. All patients known to be alive at 90 days but with missing MUAC will have an assumed change of MUAC equal to 0.
>
> Patients with unknown survival at 90 days will be assumed to be alive (with 0 change in MUAC) if allocated to intervention, or died if allocated to control. All patients known to be alive at 90 days but with missing MUAC will have an assumed change of MUAC equal to 0.

This will then give an indication of how much the results could change if the primary outcome variable were complete.

In adjusted analyses missing baseline data will be handled by multiple imputation using the MICE (multivariate imputation by chained equations) algorithm. Five multiply imputed datasets will be created and results will be combined using Rubin's rules.

8. Analysis populations

The patients will be analysed according to the initial treatment assignment, irrespective of whether the allocated treatment was received. All patients for whom the primary outcome is known will be included in the analysis, regardless of protocol adherence. Two analysis populations are defined: the intention to treat population (ITT) including all randomised patients; and the modified intention to treat (mITT) population, consisting of all randomised patients with known survival status at 90 days and (in survivors) known MUAC at baseline and 90 days.

9. Initial descriptive analyses

9.1 Recruitment, treatment allocation and follow-up

Recruitment to the Trial, treatment allocation and completeness of follow-up will be illustrated by a CONSORT flow diagram which will summarise the number of patients who were:

- Eligible and consented at admission (recruited)
- Recruited but not randomised at 48 hours (with reasons where known)
- Recruited and randomised at 48 hours
- Lost to follow-up before 90 days (with reasons)
- Known survival status but missing MUAC at 90 days
- Included in the primary analysis
- Lost to follow-up after 90 days and before 180 days (with reasons)

All participating sites have maintained a Screening Log of infants and children who are eligible (fulfil all of the inclusion criteria and none of the exclusion criteria) but not randomised, or who fulfil all of the inclusion criteria but meet one or more of the exclusion criteria. Reasons for non-recruitment will be categorised and summarised.

9.2 Baseline characteristics

The following baseline (day of admission) demographic and clinical factors will be summarised by treatment group in the ITT and mITT populations, but not subjected to statistical testing:

- Age in months – mean (sd) and median (IQR)
- Gender (male, female) – number (%)
- Weight for age Z score – mean (sd) and median (IQR)
- Height for age Z score – mean (sd) and median (IQR)
- MUAC – mean (sd) and median (IQR)
- MUAC, categorised as 11.5 to <12.5 (moderate acute malnutrition) vs >=12.5 – number (%)
- Fever (temperature >37.5°C) – number (%)
- Hypothermia (temperature <36°C)– number (%)
- Initial SpO2 – mean (sd) and median (IQR)
- Central cyanosis– number (%)
- Respiratory rate – mean (sd) and median (IQR)
- Tachypnoea – number (%)

  defined as:

Age	RR
<2 months	>=60 breaths/min
2 to 11 months	>=50 breaths/min
1 to 5 years	>=40 breaths/min
6 to 12 years	>=30 breaths/min

- Bradycardia– number (%)

  defined as:

Age	HR
<5 years	<80 bpm
≥5 years	<70 bpm

- Severe Tachycardia – number (%)

  defined as:

Age	HR
<12 months	>180 bpm
≥1 year and <5 years	>160 bpm
≥5 years	>140 bpm

- Moderate hypotension– number (%)

  defined as:

Age	SBP*
<12 months	50 to 75 mmHg
≥1 year and <5 years	60 to 75 mmHg
≥5 years	70 to 85 mmHg

* as measured with the use of an automated blood-pressure monitor

- Decompensated shock (yes, no) – number (%)

  Defined as severe hypotension (SBP<50 if age <12 months, or SBP <60 if 12 months ≤ age < 5 years, or SBP <70 if age ≥5 years)

- Compensated shock: Signs of Impaired perfusion: any one or more or capillary refill time >=2 sec, temperature gradient, weak radial pulse, severe tachycardia.
- Responsiveness (AVPU) – number (%)
- Severity of respiratory distress, assessed using an age-adjusted integrated respiratory distress and wheeze score[2] scored from 0 (no distress) to a worst possible score of 9, calculated as the sum of the following items: Audible wheeze (0-3); Ability to vocalise (0-3); Ability to feed (0-3)

Clinical history of presenting illness

- Any signs of current febrile illness (Temperature>37.5 OR temperature <36) - number (%)
- History of fever
- History of cough- number (%)
- Difficulty breathing - number (%)
- Upper respiratory tract infection (Sore throat OR Earache/ear discharge) - number (%)
- Vomiting OR Diarrhoea - number (%)
- Any history of Fits in this illness OR fitting at physical examination - number (%)

Treatment of presenting illness

- Admitted for over 24 hours in another facility - number (%)
- Oral antimalarial treatment OR any Injections or infusion of anti-malarials in the last week - number (%)
- Oral antibiotics OR injections of antibiotics - number (%)
- Type of antibiotic injections - number (%) by type
- Inhalers OR Oral steroids, all children – number (%)
- Inhalers OR Oral steroids, in children with known asthma – number (%)

Clinical examination – number (%) with each of the following:

- In-drawing
- Deep breathing
- Grunting
- Crackles /crepitations on auscultation
- Audible wheeze
- Signs of dehydration present (Sunken eyes OR Decreased skin turgor)
- Splenomegaly (gross ≥ 5 cm)
- Altered conscious level
    - Prostration (unable to sit upright)
    - Coma (unable to localise a painful stimulus or respond if <9 months)
- Suspected CNS infection (Neck stiffness or bulging fontanelle (infants only))

Past History – number (%) with each of the following:

- Gestation at birth <37 weeks
- Known HIV
- If yes, receiving antiretroviral therapy
- Previous or recent tuberculosis diagnosis

- Asthmatic (Known asthma OR Regular inhalers)
- Two or more hospital admissions in the last year
- Known epilepsy
- Previous developmental delay, defined as any one or more of the following:
  inability to walk unsupported in child aged > 18 months OR
  inability to sit unsupported in child aged > 6 months OR
  inability to suck (ever)
- Parental concerns about child's vision
- Parental concerns about child's hearing

Acute diagnosis

- Number of initial diagnoses (one vs two or more) – number (%)
- Number and % of each reported diagnosis as listed on CRF, plus number (%) with Blackwater fever (history of haemoglobinuria in this illness, or diagnosis of recurrent haemoglobinuria or dark urine syndrome).

Admission tests and microbiology results

The following admission and microbiology results will be summarised by treatment group in the ITT and mITT populations, but not subjected to statistical testing: Not all test results will be available in time for the primary analysis, these will be reported later on once available.

Haematology

- Haemoglobin - mean (sd) and median (IQR)
- Severe anaemia (Hb < 6g/dl) - number (%)
- WBC- mean (sd) and median (IQR)
- Leukocytosis (WBC > 11) - number (%)

Points of care

- Lactate > or = 5mmol/L - number (%)
- Glucose <3.0 - number (%)

Biochemistry

- Sodium- mean (sd) and median (IQR)
- Potassium- mean (sd) and median (IQR)
- Creatinine- mean (sd) and median (IQR)
- Urea- mean (sd) and median (IQR)

Malaria

- Current Malaria film positive - number (%)
- (Recent) Malaria RDT positive - number (%)

Microbiology

- Pathogen isolated - number (%)
- HIV positive - number (%)

9.3 Protocol adherence

In children randomised to RUTF, the following measures of protocol adherence will be reported:

At hospital discharge (in patient discharged before 56 days post-randomisation), number (%) of patients prescribed any RUTF, number (%) of patients prescribed RUTF to 56 days.

At 28 days follow-up, number (%) of patients still taking any RUTF; number (%) of patients still taking less than half recommended dose of RUTF; number (%) of patients still taking more than half (but not all) or recommend dose of RUTF; number (%) of patients still taking all of recommend dose of RUTF, and in those not still taking RUTF, mean (sd) number of missed days, and reasons for stopping.

9.4 Withdrawals

The number and percentage of patients with written consent at or after randomisation will be reported, by treatment group. Following randomisation, the number and percentage of patients, by arm, in the following groups will be summarised:

Consent never obtained, died without consent

Consent never obtained, absconded without consent

Consent declined (with reasons if known)

Withdrew following consent (with reasons if known)

10.Primary analysis

The primary outcome will be compared between arms using a Wilcoxon rank-sum test with two-sided alpha of 0.05. The primary effect estimate will be the probabilistic index (the probability that the intervention is superior to the control for either mortality and/or change in MUAC), which will be presented with a 95% confidence interval. The individual components of the composite endpoint (change in MUAC in patients surviving to day 90, and mortality by day 90) will be additionally reported and compared between arm as follows:

In all patients surviving to day 90- with non-missing MUAC, the unadjusted mean difference between arms in MUAC change from baseline will be presented with a 95% confidence interval. A generalized linear model will be fitted to compare change from baseline after adjusting for arm, baseline MUAC, and trial site (as a random factor).

In all randomised patients, survival to 90 days will be compared between arms using Cox regression with patients who were alive at their 90 days visit censored at the earlier of 90 days or last known date of follow-

up. The regression model will include arm, baseline MUAC, and trial site (using shared frailty), and will be used to calculate an adjusted hazard ratio with 95% confidence interval.

11.Secondary analyses

Binary outcomes (disability free survival to 28 days, re-admission to hospital by 20 and 180 days, resolution of neurocognitive sequelae) will be reported in each treatment group as counts and percentages. Absolute risk reduction and unadjusted odds ratios will be reported with 95% confidence intervals. Multilevel logistic regression (adjusted for treatment group, baseline MUAC, and trial site (as a random factor) will be used to calculate adjusted odds ratios with 95% confidence intervals. Only patients surviving to the relevant follow-up visit will be included in these analyses.

MUAC, TST and weight and height Z-scores will be reported for surviving patients at 28, 90 and 180 days in each treatment group. Unadjusted mean difference between groups will be calculated, with 95% confidence intervals. Linear regression (adjusted for treatment group, baseline measurement, and trial site (as a random factor)) will be used to calculated adjusted mean differences between groups.

Survival will be described using Kaplan-Meier survival curves by treatment group, and unadjusted and adjusted hazard ratios with 95% confidence intervals will be calculated using Cox regression models (adjusted for treatment group, baseline MUAC, and trial site (using shared frailty). Surviving patients will be censored at date of last known follow-up. Estimated survival probability at 28 and 180 days (with 95% confidence intervals) will be reported by treatment group.

12.Subgroup analyses

Hypothesis generating analyses will be performed to explore the potential impact of various baseline clinical factors on MUAC change by 90 days in surviving patients, and on overall survival (if the total number of events allows for this).

For each outcome, the adjusted treatment effect will be calculated as described previously, but with the regression model also including the following additional variables which are measured in the first hospital admission.

Presence of fever (defined as temperature>37.5 or < 36 ^{o}C at screening);

malaria (defined as positive RDT or positive blood film on admission)

Any microbiological evidence of sepsis (blood culture or retrospective molecular diagnosis) ;

radiographic evidence of pneumonia (signs recorded in right and/or left lung on chest X-ray consistent with pneumonia);

HIV;

severe anaemia (haemoglobin <5g/dl);

*Sickle cell disease (known or diagnosed by PCR on admission sample)

*to be included if results are available from blood & urine laboratory analysis, as these are to be perfomed retrospectively for research purposes only and are not available during the hospital admission.

For each variable In turn, an interaction term between the subgroup variable and treatment allocation will be tested for significance, and an estimated odds ratio with 95% confidence interval will be calculated for each subgroup.

13. Interim analyses

One interim analysis was planned following randomisation of 420 patients. Guidelines to recommend early termination were based on a Peto-Haybittle stopping rule ($P<0.001$).

The interim analysis was reported to the DMEC on 13th September 2021 who recommended that the trial continue as planned.

14.Timing of primary analysis

The primary clinical endpoint and all secondary clinical endpoints will be analysed after all patients have completed their 120 day follow-up. At this point all planned analysis not involving any pending laboratory results will be performed A further analysis of extra baseline characteristics and subgroups to include all biochemistry data will be performed once this data is available, but any analysis which had been run previously will not be repeated at this stage.

15.Safety

The number and percentage of Serious Adverse Events (SAEs) within 180 days following randomisation will be reported by treatment group. The types of SAEs, whether they were fatal or not and the perceived relatedness to RUTF will be summarised by treatment group. Cause of death will be adjudicated by an Endpoint Review Committee (ERC) blinded to randomised allocations. The total number and percentage of allergic reactions to RUTF (whether or not these were additionally reported as SAEs) will be reported.

16.Statistical software

All analyses will be conducted in Stata/SE Version 16.1 64-bit x86-64 (StatCorp LLC, College Station, TX).

References

[1] Gamble C, Krishan A, Stocken D, et al. Guidelines for the Content of Statistical Analysis Plans in Clinical Trials. JAMA 2017;318:2337-43.

[2] Liu LL, Gallaher MM, Davis RL, Rutter CM, Lewis TC, Marcuse EK. Use of a respiratory clinical score among different providers. Pediatric pulmonology. 2004; 37(3): 243-8.
